# Combined Analysis of Transcriptomes and Metabolomes Reveals That MeJA-Mediated Flavonoid Biosynthesis Is Crucial for Pigment Deposition in Naturally Colored Green Cotton Fibers

**DOI:** 10.3390/genes16050599

**Published:** 2025-05-19

**Authors:** Shuangquan Xie, Kailu Chen, Rui Tang, Xuechi Li, Yuxin Wei, Yijie Cheng, Shouwu Tang, Wengang Chen, Quanliang Xie, Zhuang Meng, Asigul Ismayil, Xiang Jin, Fei Wang, Haifeng Liu, Hongbin Li

**Affiliations:** 1 Key Laboratory of Xinjiang Phytomedicine Resource and Utilization of Ministry of Education, College of Life Sciences, Shihezi University, Shihezi 832000, China; xiesq@shzu.edu.cn (S.X.); ckl3224832883@163.com (K.C.); tr2604791944@163.com (R.T.); 18699318595@163.com (X.L.); 13629936504@163.com (Y.W.); yijiecheng803@163.com (Y.C.); xiequan-liang001@shzu.edu.cn (Q.X.); zhuangmeng610@163.com (Z.M.); asgli12@163.com (A.I.); feiw@shzu.edu.cn (F.W.); 2 China Colored-Cotton (Group) Co., Ltd., Urumqi 830000, China; shouwutang@126.com (S.T.); chen-wengang0917@163.com (W.C.); 3 Ministry of Education Key Laboratory for Ecology of Tropical Islands, College of Life Sciences, Hainan Normal University, Haikou 570000, China; jinx@hainnu.edu.cn

**Keywords:** green cotton fiber, pigment deposition, transcriptome, metabolome, MeJA, flavonoid biosynthesis

## Abstract

**Background**: Green cotton fibers (GCFs) are valued for their natural coloration and eco-friendly properties, but their pigmentation mechanisms remain unclear, limiting their wider application in the textile industry. This study aims to uncover the key regulatory genes and metabolic pathways involved in GCF coloration. **Methods**: We conducted transcriptome and metabolome profiling of green and white cotton fibers at different developmental stages to identify differences in gene expression and metabolite accumulation related to pigmentation. **Results**: Transcript analysis revealed significant enrichment in α-linolenic acid metabolism, flavonoid biosynthesis and phenylpropane metabolism pathways during late pigmentation stages. Key genes in methyl jasmonate (MeJA) biosynthesis and flavonoid biosynthesis (*LOX*, *JMT*, *ANS*, *C4H*, *DFR*, *F3H*) were upregulated. The MYB transcription factor showed the most significant increase during fiber development. Metabolomic analysis identified 12 metabolites that accumulated specifically in green fibers. MeJA treatment promoted the expression of MYB genes and flavonoid biosynthesis genes (*DFRs*, *ANSs*, *F3H*, C4H), as well as the accumulation of Luteolin, Gallocatechin, Cyanidin and Chrysanthemum metabolites. **Conclusions**: Our study demonstrates that MeJA-mediated flavonoid biosynthesis, regulated by MYB transcription factors, is the central pathway controlling pigment deposition in GCFs. These findings provide valuable insights for developing improved colored cotton materials.

## 1. Introduction

Natural colored cotton (NCC) represents a biologically pigmented variant of *Gossypium hirsutum*, and green and brown cottons are two major cultivars for their fibers utilized for the textile industry [1]. In the current cotton fiber-processing methods, dyes are often used for coloration. In contrast, naturally colored cotton fibers avoid this step, thereby reducing environmental pollution and health hazards [2]. These colored cotton cultivars exhibit advantages of both prominent resistance to stress and eco-friendly quality, disease resistance, salt tolerance, and drought tolerance [3,4]. However, the instability of NCC pigment deposition greatly limits its utilization in related industrial fields [5]. Thus, understanding and elucidating the mechanism of pigment deposition is the key issue in NCC application.

As one of the major NCC cultivars, green cotton holds an important position in colored fiber production [6,7]. Compared to brown cotton, research on green cotton is relatively less reported [8]. Overexpression of the *anthocyanidin 3-O-glucosyltransferase* (*3GT*) gene in brown cotton varieties results in a green cotton phenotype, likely due to the upregulation of *Gh3GT* leading to increased anthocyanin levels, redirecting the metabolic flux towards anthocyanins [9,10]. Conversely, silencing the *chalcone isomerase* (*CHI*) gene in brown cotton using RNAi technology produces three color variants: light brown, green, and nearly white [9]. Studies have indicated that the functional involvement of the *4-coumarate: CoA ligase* (*4CL*) gene in the natural pigmentation process of green cotton fibers [11], while inhibiting *phenylalanine ammonia-lyase* (*PAL*) enzyme activity, results in white fibers in vitro [12]. Other flavonoid synthesis-related genes, such as *chalcone synthase* (*CHS*), *flavanone 3-hydroxylase* (*F3H*), *flavonoid 3′-hydroxylase* (*F3′H*), *flavonol synthase* (*FLS*), and *leucoanthocyanidin reductase* (*LAR*), are significantly upregulated in green cotton fibers, generating the conclusion that flavonoid metabolites are important components of green cotton pigments, primarily consisting of colorless anthocyanins, flavonols, and flavanols [11,13].

Plant hormones orchestrate crucial physiological processes ranging from cell division to organogenesis and stress responses. Methyl jasmonate (MeJA), which serves as a functional phytohormone, is involved in stress responses. Previous studies have demonstrated that preharvest application of methyl jasmonate (MeJA) promotes fruit ripening, enhances color development, and improves antioxidant properties in ‘Yoho’ and ‘Jiro’ persimmons [14,15]. In vitro studies have demonstrated that while low concentrations (0.05 μM) of jasmonic acid (JA) promote cotton fiber elongation, this stimulatory effect diminishes at 0.1 μM, and higher concentrations (>0.5 μM) of MeJA completely inhibit elongation [16,17]. Evidence has shown that MeJA induced flavonoid biosynthesis in various plants, including apple (*Malus domestica*) [18], grape (*Vitis vinifera*) [19], blueberry (*Vaccinium corymbosum*) [20], and strawberry (*Fragaria x ananassa*) [21]. Transcription factors MYB and bHLH, as key regulators of flavonoid biosynthesis pathway genes, are also regulated by MeJA [22,23]. In Caitai, MeJA mediated tissue-specific anthocyanin accumulation by affecting flavonoid metabolic pathway genes [24]. In pear, MeJA promoted accumulation of flavonoid metabolites by upregulating flavonoid biosynthesis pathway genes, including *CHS*, *CHI*, *F3H*, *anthocyanidin synthase* (*ANS*), *leucoanthocyanidin reductase* (*LAR*), and *dihydroflavonol 4-reductase* (*DFR*) [25].

Although studies have reported genes and metabolites related to pigment deposition in green cotton fibers, the regulatory mechanism remains unclear, and the involvement and function of plant hormones in this process are still unknown. In this investigation, we performed a conjoint analysis of transcriptome data at both developmental stage dimensions of GCFs and comparative dimensions between GCFs and white fibers, and of targeted metabolomics data. We observed that in flavonoid and MeJA biosynthesis pathways, *MYBs* and flavonoid biosynthesis pathway genes were significantly enriched in GCFs, and flavonoid biosynthesis pathway metabolites were also significantly accumulated in GCFs. Exogenous MeJA significantly promoted the expression of *MYBs* and flavonoid biosynthesis pathway genes and the corresponding metabolites. Our research provides important insights and guidance for interpreting the molecular mechanism of pigment deposition in GCFs and for cultivating excellent green cotton materials through genetic engineering techniques.

## 2. Results

### 2.1. Comparative Analysis of the Transcriptomes Between Green and White Fibers

**Figure 1:**
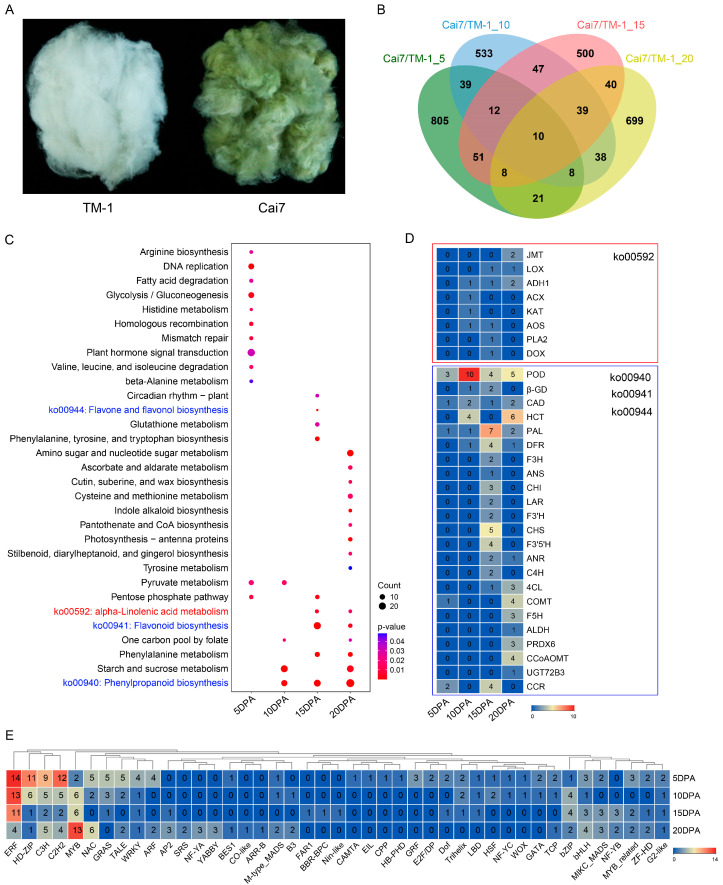
Analysis of comparative transcriptomes between green fibers (Cai7) and white (TM-1) at different development stages (0, 5, 10, 15, and 20 days post anthesis, DPA). (**A**) The mature fiber phenotypes of green cotton Cai7 and white cotton. (**B**) Venn diagram of the upregulated differentially expressed genes (UDEGs) in Cai7 relative to TM-1 in 5-, 10-, 15-, and 20-DPA fibers. (**C**) KEGG enrichment analysis of the UDEGs in 5-, 10-, 15-, and 20-DPA fibers. Red and blue texts indicate the methyl jasmonate (MeJA) synthesis pathway and flavonoid-related pathway, respectively. (**D**) Quantitative analysis of UDEGs associated with MeJA synthesis and flavonoid-related pathway. Red and blue frames show the MeJA synthesis pathway and flavonoid-related pathway, respectively. (**E**) Statistical identification of UDEGs related to different family members of transcription factors (TFs). The cluster analysis was carried out based on a hierarchical clustering algorithm; the Euclidean distance was chosen for the distance measurements, and the fully connected method was adopted for the final cluster association determination.

We identified fibers of green cotton Cai7 and white cotton TM-1 (Figure 1A) for comparative transcriptomic analysis at five stages of fiber maturation. Compared to white fibers, green fibers had 954, 726, 707, and 863 upregulated differentially expressed genes (UDEGs) at periods of 5, 10, 15, and 20 days post anthesis (DPA), respectively. The Venn diagram results showed that 2850 UDEGs and 10 co-expressed UDEGs (CUDEGs) were identified across the four periods (Figure 1B). KEGG analysis indicated significant enrichment of twelve, four, nine, and fifteen pathways in the four periods, respectively, with eight pathways enriched in more than one period. Interestingly, in the last two periods of fiber development (15 DPA and 20 DPA), pathways of α-linolenic acid metabolism and flavonoid biosynthesis were significantly enriched. Phenylpropanoid biosynthesis was considerably enriched in three periods (10, 15, and 20 DPA) (Figure 1C). In the α-linolenic acid metabolism pathway, eight gene family members, including *jasmonic acid carboxyl methyltransferase* (*JMT*), *lipoxygenase* (*LOX*), *alcohol dehydrogenase 1* (*ADH1*), *acyl-CoA oxidase* (*ACX*), *3-ketoacyl-CoA thiolase* (*KAT*), *allene oxide synthase* (*AOS*), *phospholipase A2* (*PLA2*), and *fatty acid α-dioxygenase* (*DOX*), were discovered (Figure 1D and Figure S1). This significantly enriched pathway and its associated genes indicate a regulatory role for MeJA-mediated signaling in controlling pigment deposition in GCFs. Besides the pathways of phenylpropanoid and flavonoid biosynthesis, significant enrichment was observed for flavone and flavonol biosynthesis pathways at 10 DPA, with 23 related gene family members detected, including *peroxidase* (*POD*), *hy-droxycinnamoyl-CoA*: *shikimate hydroxycinnamoyl transferase* (*HCT*), *PAL*, and *CHS* gene families (Figure 1D and Figures S2–S4). Transcription factor analysis revealed that all UDEGs contained 43 types of TFs, with ERF, MYB, HD-ZIP, C3H, and C2H2 being the most significant accumulated TFs. These results suggest that MeJA, flavonoid biosynthesis, and TFs may perform important roles in pigment deposition of GCFs.

### 2.2. Transcriptomic Profiling of GCFs Across Developmental Stages

**Figure 2:**
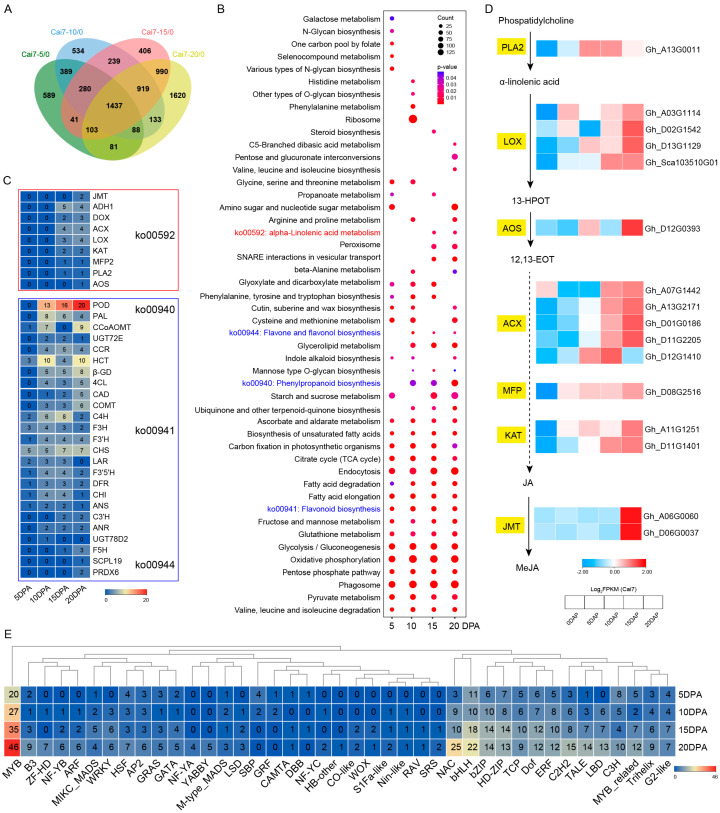
Transcriptomic profiling of Cai7 fibers across developmental stages. (**A**) Venn plots of differentially expressed genes upregulated at 5, 10, 15, and 20 DPA relative to 0 DPA in Cai7 fibers. (**B**) KEGG enrichment analysis of the upregulated genes (UDEGs) at 5 DPA, 10 DPA, 15 DPA, and 20 DPA relative to 0 DPA in Cai7 fibers. Red and blue texts represent the MeJA synthesis pathway and flavonoid-related pathway, respectively. (**C**) Statistical analysis was performed on the number of UDEGs corresponding to different family members associated with MeJA synthesis and flavonoid-related pathways. Red and blue frames delegate the MeJA synthesis pathway and flavonoid-related pathway, respectively. (**D**) Expression-level heatmap of UDEGs located in the MeJA synthesis pathways at different development periods of Cai7 fibers. (**E**) Quantitative assessment of UDEG counts across transcription factor (TF) family representatives. The hierarchical clustering method based on Euclidean distance and the complete link method are used for clustering analysis.

During different development periods of GCFs, 7849 genes were identified as UDEGs compared to 0 DPA, including 1437 CUDEGs. At 5, 10, 15, and 20 DPA, there were 3008, 4119, 4415, and 5371 UDEGs, respectively (Figure 2A). KEGG analysis showed that fatty acid-related pathways (fatty acid degradation, fatty acid elongation, and biosynthesis of unsaturated fatty acids), sugar-related pathways, starch and sucrose metabolism, mannose type O-glycan biosynthesis, fructose and mannose metabolism, and redox-related pathways (peroxisome and oxidative phosphorylation) were significantly enriched. Interestingly, four pathways, including α-linolenic acid metabolism, phenylpropanoid biosynthesis, flavonoid biosynthesis, and flavone and flavonol biosynthesis, were also significantly enriched during green cotton fiber development (Figure 2B), indicating high consistency with the results of comparative transcriptomic data between white and green fibers (Figure 1). Additionally, almost all MeJA synthesis genes such as *PLA2*, *LOX*, *AOS*, *ACX*, *multifunctional enzyme* (*MFP*), *KAT*, and *JMT* were identified in the α-linolenic acid metabolism pathway, especially in the latter two periods of fiber development (15 and 20 DPA) (Figure 2C,D and Figure S5), showing the potential important role of MeJA in regulating green cotton fiber development. Flavonoid synthesis pathway genes, including *POD*, *PAL*, *HCT*, *CHS*, *caffeoyl-CoA O-methyltransferase* (*CCoAOMT*), and *β-glucosidase* (*β-GD*), were identified as the most significant abundant family members (Figure 2C and Figures S6–S8). Transcription factor analysis results showed that UDEGs contained a large number of TFs that were significantly accumulated in GCFs, especially MYBs, the most abundant genes in GCFs (Figure 2E). These results indicate that MeJA synthesis, flavonoid synthesis, and TF MYBs might be the crucial factors involved in fiber development and pigment deposition in GCFs.

### 2.3. Multi-Dimensional Conjoint Analysis of White and Green Fibers Transcriptomes

**Figure 3:**
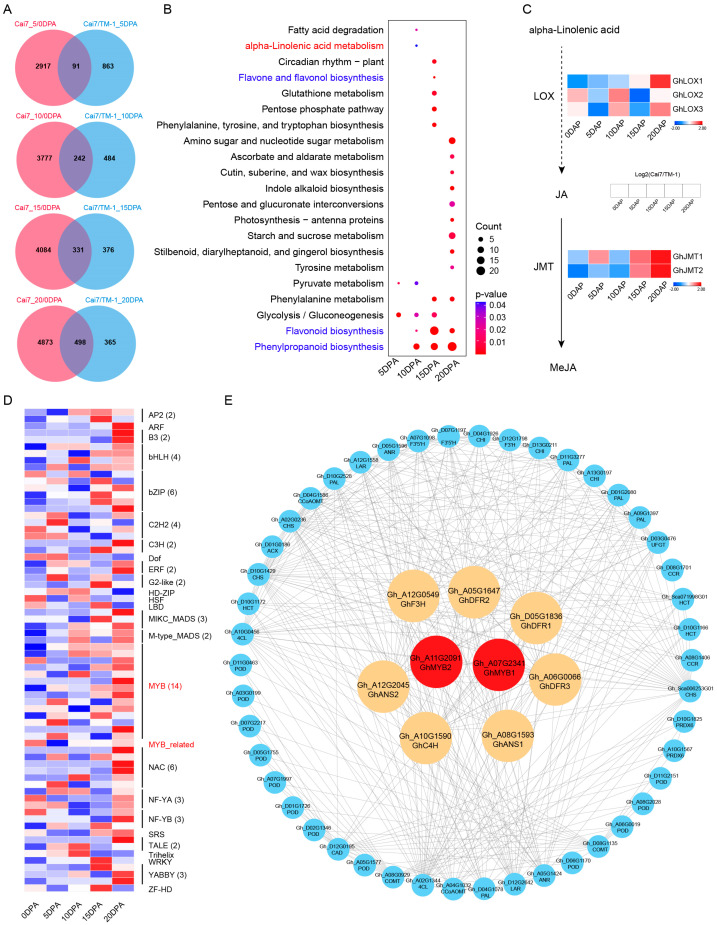
Comparative multi-dimensional transcriptomics of Cai7 and TM-1 fiber developmental trajectories. (**A**) UDEG overlaps in Cai7 and TM-1 fibers at 5–20 DPA (Venn diagram). The intersection of each period is defined as co-expressed UDEGs (CUDEGs). (**B**) KEGG enrichment analysis of CUDEGs at 5, 10, 15, and 20 DPA in Cai7 and TM-1 fibers. Red and blue texts show the MeJA synthesis pathway and flavonoid-related pathway, respectively. (**C**) Heatmap visualization of expression profiles of CUDEGs located in the MeJA synthesis pathway in Cai7 fibers relative to TM-1 fibers at different fiber development periods. (**D**) TF expression-level heatmap of the of Cai7 relative to TM-1 at different fiber development periods. (**E**) PPI analysis of the CUDEGs distributed in the flavonoid-related pathway and TFs in different development periods. Red, orange, and blue colors represent TFs, direct interaction genes with TFs predicted by PPI, and other flavonoid-related pathway CUDEGs, respectively. The clustering analysis employed hierarchical clustering using Euclidean distance and the complete linkage method.

To further investigate the key pathways and genes possibly regulating green fiber development and pigment deposition, we conducted a multi-dimensional comprehensive analysis of the transcriptomes of both green fibers during different developmental stages and of the comparative transcriptomes between white and green fibers. Analysis revealed that exactly 91, 242, 331, and 498 CUDEGs were identified as the co-expressed genes in four periods, respectively (Figure 3A). KEGG analysis indicated that phenylpropanoid biosynthesis and flavonoid biosynthesis pathways revealed significant enrichment during the latter three stages of fiber development (10, 15, and 20 DPA) (Figure 3B). Additionally, α-linolenic acid metabolism and the biosynthesis of flavones and flavonols were also enriched, in which five genes from two family members participated in MeJA synthesis, with *GhLOX1*, *GhLOX2*, and *GhLOX3* from the LOX family, and *GhJMT1* and *GhJMT2* from the JMT family (Figure 3C). TF analysis showed that CUDEGs included 70 TF genes from 25 families including 15 *MYBs* (Figure 3D). Protein–protein interaction (PPI) analysis of transcription factors and the regulated genes from the aforementioned four pathways showed that two MYB transcription factors, *GhMYB1* and *GhMYB2*, had direct interactions with seven flavonoid pathway genes of *GhF3H*, *GhDFR1*, *GhDFR2*, *GhDFR3*, *GhANS1*, *GhANS2*, and *GhC4H* (Figure 3E).

**Figure 4:**
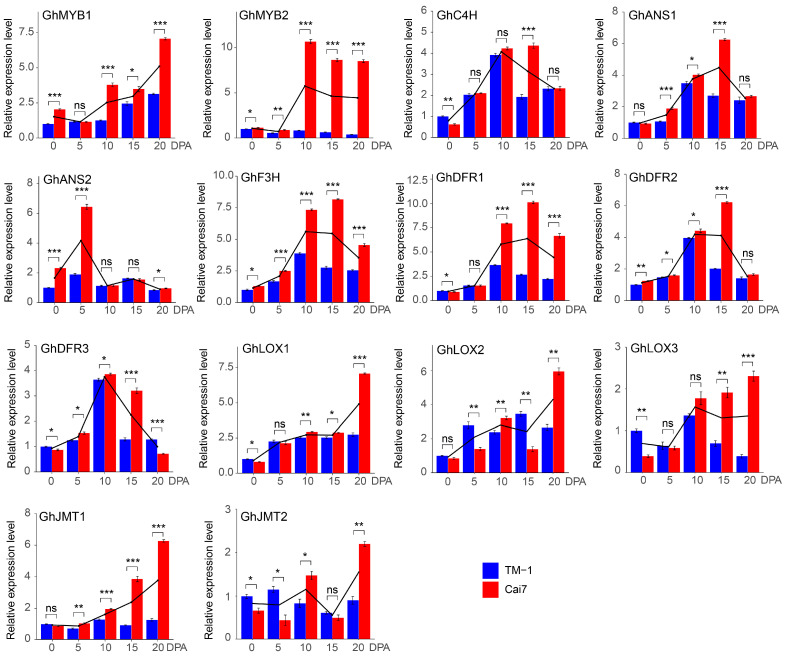
RT-qPCR was performed to verify candidate genes of TFs, MeJA synthesis, and flavonoid-related pathways. The genes of TFs (*GhMYB1* and *GhMYB2*), flavonoid-related pathway genes (*GhC4H*, *GhANR1-GhANR2*, *GhF3H*, and *GhDFR1-GhDFR3*) and MeJA synthesis pathway genes (*GhLOX1-GhLOX3* and *GhJMT1-GhJMT2*) analyzed with RT-qPCR using the materials of 0-, 5-, 10-, 15-, and 20-DPA ovules and fibers of Cai7 and TM-1. Black lines represent the trend of gene expression levels in Cai7 and TM-1 fibers at different stages of development. Statistical significance was determined using a *t*-test, and the significance level was set as * *p* less than 0.05, ** *p* less than 0.01, and *** *p* less than 0.001, and ns means not significant.

To validate the transcriptome data accuracy, expression profiles of the representative candidate genes were detected using RT-qPCR, and comparative analysis revealed a high consistency between the transcriptome data and the RT-qPCR results (Figure 4). These experimental observations indicate that MeJA, flavonoid synthesis, and the two MYBs might be involved in pigment deposition in GCFs.

### 2.4. Comparative Metabolomic Profiling of White Versus Green Fibers

**Figure 5:**
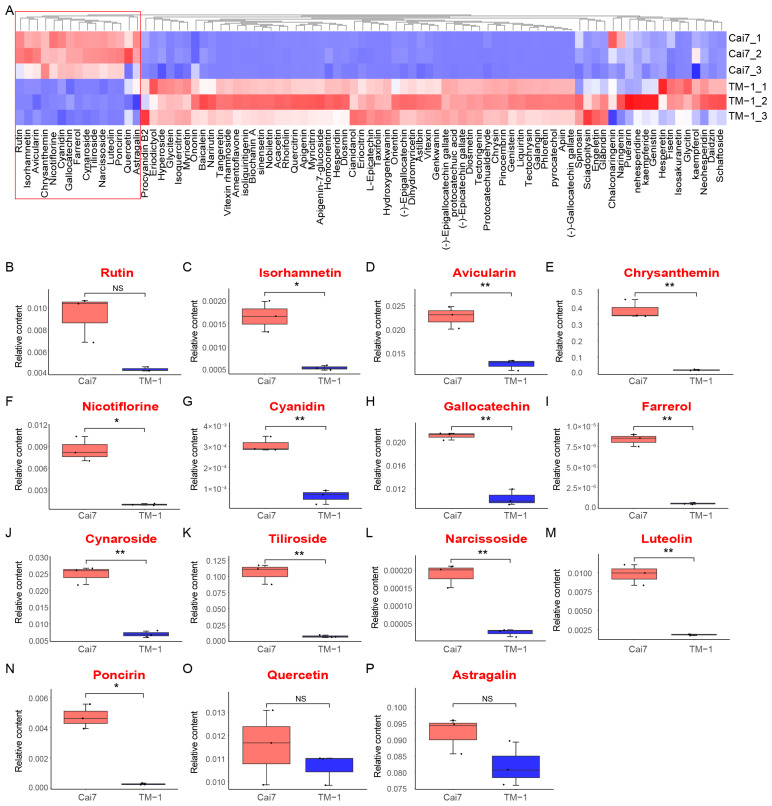
Quantitative profiling of flavonoid metabolite content levels in Cai7 and TM-1 fibers through extensive targeted metabolomics. (**A**) Comparative heatmap clustering of all detected flavonoid metabolites. Clustering analysis was conducted using hierarchical clustering using Euclidean distance and the complete linkage method. (**B**–**P**) Boxplot of six significantly upregulated metabolites in fibers of Cai7 relative to TM-1. Significance level was set as * *p* less than 0.05, ** *p* less than 0.01, and *** *p* less than 0.001, and NS means not significant.

Regarding the transcriptomic results of flavonoid synthesis as the significantly enriched pathway in GCFs, we performed a broad-targeted comparative analysis of flavonoid metabolites between white and green fibers. Principal component analysis delineated a visible segregation between green and white fiber samples (Figure S9), and 85 flavonoid metabolites were identified in total. (Figure 5). Hierarchical clustering analysis of relative metabolite content and volcano plot analysis from OPLS-DA (Orthogonal Projections to Latent Structures Discriminant Analysis) demonstrated that 15 flavonoid metabolites were upregulated in GCFs compared to white fibers. In total, 12 metabolites including Isorhamnetin, Poncirin, Avicularin, Chrysanthemin, Nicotiflorin, Cyanidin, Gallocatechin, Farrerol, Cynaroside, Tiliroside, Narcissoside, and Luteolin were specifically significantly upregulated in GCFs (Figure 5). These results suggest that these 12 flavonoid pathway metabolites might be important regulators of pigment deposition in GCFs.

### 2.5. Combined Analysis of Transcriptome and Metabolome in White and Green Fiber Variants

**Figure 6:**
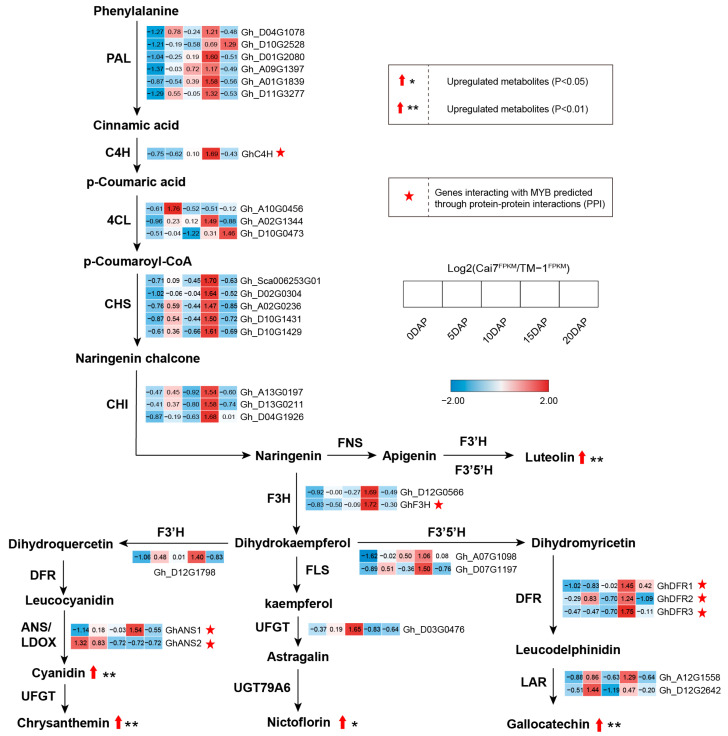
Quantitative analysis of flavonoid content in Cai7 and TM-1 fibers using targeted metabolomic technology. Heatmap and cluster analysis were performed on all detected flavonoid metabolite content levels, and hierarchical cluster analysis was carried out using Euclidean distance and complete connection methods. The boxplot showed six metabolites that were significantly upregulated in Cai7 fibers relative to TM-1. Significance level was set as * *p* less than 0.05, ** *p* less than 0.01, and *** *p* less than 0.001, and NS means not significant.

To study the correlation between differentially expressed genes (UDEGs) and flavonoid metabolites, we analyzed correlations between the UDEGs in the flavonoid synthesis pathway in the transcriptome and 12 flavonoid metabolites with notable accumulation. The results show that five flavonoid metabolites, Luteolin, Gallocatechin, Nicotiflorin, Cyanidin, and Chrysanthemin, as well as the metabolite-encoding genes *GhC4*, *GhF3H*, *GhDFR1/2/3*, and *GhANS1/2,* which are regulated by MYBs, indicated similar accumulated levels (Figure 6). Of these, *GhC4H* was upstream of the synthesis of all five metabolites and was involved in their production. *GhF3H* participated in the synthesis of all five metabolites except Luteolin. *GhDFR1/2/3* genes were closely related to the generation of Cyanidin, Chrysanthemin, and Gallocatechin, and *GhANS1/2* genes were directly associated with the synthesis of Cyanidin and Chrysanthemin (Figure 6). These results suggest a direct or indirect regulatory network among MYBs, genes of the flavonoid biosynthetic pathway, and flavonoid metabolites for controlling pigment deposition in GCFs.

### 2.6. Changes in Flavonoid Pathway Gene Expression Levels and Metabolite Contents in GCFs Under Exogenous MeJA Treatment

**Figure 7:**
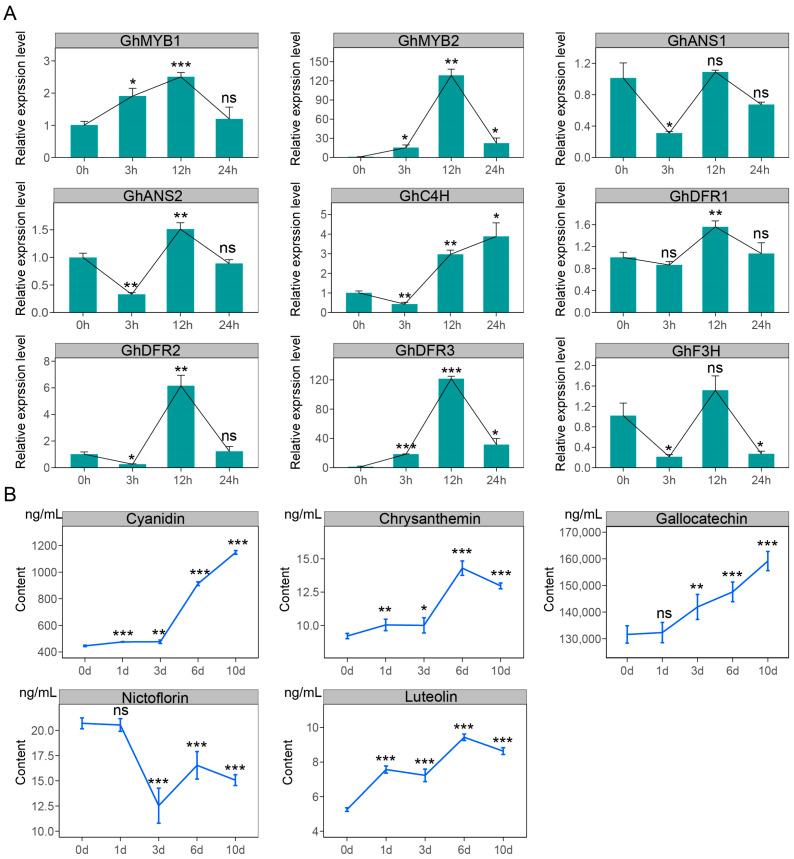
Transcriptional reprogramming of MYB transcription factors and flavonoid biosynthesis genes coupled with flavonoid accumulation in green cotton fibers treated with MeJA. The 1-DPA ovules of Cai7 were collected from cotton bolls and cultured on BT medium for 7 days, followed by treatment with 5 µM MeJA. The treated ovules and fibers were subsequently subjected to comprehensive analysis. (**A**) The expression levels of GhANS1/2, GhDFR1/2/3, GhC4H, and GhF3H were detected in Cai7 ovules and fibers treated for 0, 3, 12, and 24 h with MeJA. Black lines represent the trend of gene expression levels during different treatment time points. (**B**) The contents of Luteolin, Gallocatechin, Cyanidin, Nicotiflorin, and Chrysanthemin were determined in Cai7 ovules and fibers treated for 0, 1, 3, 6, and 10 days with MeJA. Blue lines denote the trend of content changes. Statistical significance was determined using a *t*-test, and the significance level was set as * *p* less than 0.05, ** *p* less than 0.01, and *** *p* less than 0.001, and ns means not significant.

To evaluate the function of MeJA in green cotton fiber development, cotton ovules were collected 1 day after anthesis (1 DPA), cultured for 7 days, and then treated with MeJA at different times, including 0, 3, 12, and 24 h. The treated ovules’ associated fibers were used to perform RT-qPCR analysis of two MYB transcription factors and seven flavonoid-related candidate genes. The results showed that, except for *GhF3H* and *GhANS1*, the remaining genes indicated significantly induced expressions by MeJA. Notably, the two MYB transcription factors *GhMYB1* and *GhMYB2* exhibited sustained high expression from 0 to 12 h, and the other five pathway genes showed significantly increased expression levels at 12 h (Figure 7A). These observations suggest that MeJA significantly induces the expression patterns of genes related to green cotton fiber development. The MeJA-treated ovules’ associated fibers at different time points of 0, 1, 3, 6, and 10 days were also collected for content detection of five flavonoids in green cotton fibers using HPLC-MS/MS. The findings revealed that, after MeJA treatment, except Nicotiflorin, which showed a decreased tendency, the other four flavonoid metabolites, Luteolin, Gallocatechin, Cyanidin, and Chrysanthemin, were significantly accumulated (Figure 7B). These findings suggest that MeJA, as a key regulatory factor, promotes the synthesis of flavonoid metabolites by modulating the expressions of associated genes and TFs in green cotton fibers.

## 3. Discussion

### 3.1. α-Linolenic Acid Metabolism and Flavonoid-Related Pathways Are Significantly Enriched During Green Cotton Fiber Development

The development of colored cotton fibers is generally consistent with that of white-fiber cotton, going through five stages: cell initiation (0–3 DPA), cell elongation (3–16 DPA), secondary wall thickening (16–20 DPA), cell wall thickening (20–40 DPA), and maturation (40–50 DPA) [26,27,28,29,30,31,32,33,34]. The key difference is that colored cotton fibers undergo a pigment synthesis and deposition process, occurring at approximately 15–25 DPA [1,9]. In this study, by comparing the transcriptomes of green and white cotton fibers at 5, 10, 15, and 20 DPA, we found the significant enrichment of the α-linolenic acid metabolism and flavonoid-related pathways (phenylpropanoid biosynthesis, flavonoid biosynthesis, and flavone, and flavonol biosynthesis) specifically in 15- and 20-DPA GCFs (Figure 1C). Transcriptomic analysis of different periods of green cotton fiber development also showed highly consistent results (Figure 2B). The enrichment results for flavonoid-related pathways are similar to those of previous studies. In plants, the primary pathway for pigment synthesis is the flavonoid biosynthesis pathway, which is closely related to the color of wheat grains, sunflower and tulip flowers, and brown cotton fibers [1,23,29]. Comprehensive transcriptome analysis of gene expression profiles of green and white cotton fibers at different stages of development further confirmed the close relationship between pigment deposition in green cotton fibers and flavonoid biosynthesis pathways (Figure 3B). Similarly, transcriptome analyses from different dimensions consistently showed significant enrichment in the α-linolenic acid metabolism pathway (Figure 1C, Figure 2B and Figure 3B), which is involved in the synthesis of MeJA, suggesting that MeJA may participate in the pigment deposition process in GCFs. Evidence has shown that the addition of a methyl jasmonate synthesis inhibitor (SHAM) results in a lighter color of brown cotton fibers and decreased fresh weight of ovules, dry weight of fibers, and dry weight of ovules, which indirectly supports the key role of MeJA in the development of colored cotton fibers and pigment synthesis [30].

### 3.2. MeJA Plays a Regulatory Role in Green Cotton Fiber Development and Pigmentation

Numerous reports have established a strong connection between MeJA and the synthesis of flavonoid compounds. Research has indicated that the flavonoid content in *Scutellaria baicalensis*, apple, grape, blueberry, and strawberry significantly increased following MeJA treatment [18,19,20,21,31]. Our results also showed that genes related to MeJA synthesis are specifically significantly enriched in developing green cotton fibers (Figure 2D, Figures S1 and S5). Multi-dimensional analysis revealed that the genes *GhLOX1/2/3* and *GhJMT1/2* are significantly increased in GCFs as important candidates for MeJA synthesis (Figure 3C and Figure 4). Earlier investigations revealed that MeJA played a key role in the elongation and pigment accumulation of brown cotton fibers [30]. Additionally, in plants like Caitai and pear, MeJA has been shown to regulate the formation and accumulation of flavonoid compounds [24,25]. In this study, the contents of flavonoids such as Luteolin, Gallocatechin, Cyanidin, and Chrysan-themin showed significant upregulation in green cotton fibers treated with MeJA (Figure 7B). Based on these findings, we can infer that MeJA performs a role in the development and pigment deposition of green cotton fibers by modulating the levels of flavonoid pathway-related genes and metabolites.

### 3.3. Flavonoid Biosynthesis Genes and MYB Factors Synergistically Control Green Pigmentation of Cotton Fibers

Investigations have indicated that flavonoid biosynthetic genes are a key class of genes involved in pigment synthesis in plants [23]. CHS catalyzes the formation of chalcone, a major precursor of flavonoid compounds [11]. Genes of *F3′H* and *F3′5′H* were crucial for the synthesis and accumulation of anthocyanins [32]. In colored cotton, the transcriptome and protein expression levels of *GhANR1/2* and *GhANS* in brown cotton fibers at 15 DPA were significantly increased compared to white cotton, with genes such as *PAL*, *CHS*, *F3H*, *DFR*, and *UFGT* leading to pigment synthesis and deposition through the synthesis of related flavonoid metabolites [1,32,34]. The *Gh4CL* gene is involved in the coloration of green cotton fibers [11]. In our work, pathways related to flavonoid synthesis were significantly enriched in the pigment deposition of GCFs (Figure 1C, Figure 2B and Figure 3B). Many flavonoid synthesis genes were specifically significantly upregulated in GCFs (Figure 1D, Figure 2C, Figures S2–S4 and S6–S8), including various family members of *F3H*, *DFR*, *ANS*, and *C4H*. TF analysis revealed that MYBs constitute the core regulatory network controlling GCF morphogenesis (Figure 3D). PPI analysis suggested that two MYBs (GhMYB1 and GhMYB2) might have a direct regulatory link with *GhF3H*, *GhDFR1/2/3*, *GhANS1/2*, and *GhC4H* (Figure 3E). MYBs have been reported as the most common TFs in anthocyanin synthesis [23], and they participate in pigment synthesis in brown cotton fibers by regulating the expression of *ANS*, *ANR*, *UFGT*, and *F3H* [1].

### 3.4. MeJA–MYB–Flavonoid Regulatory Network Promotes the Pigmentation of Green Cotton Fiber

**Figure 8:**
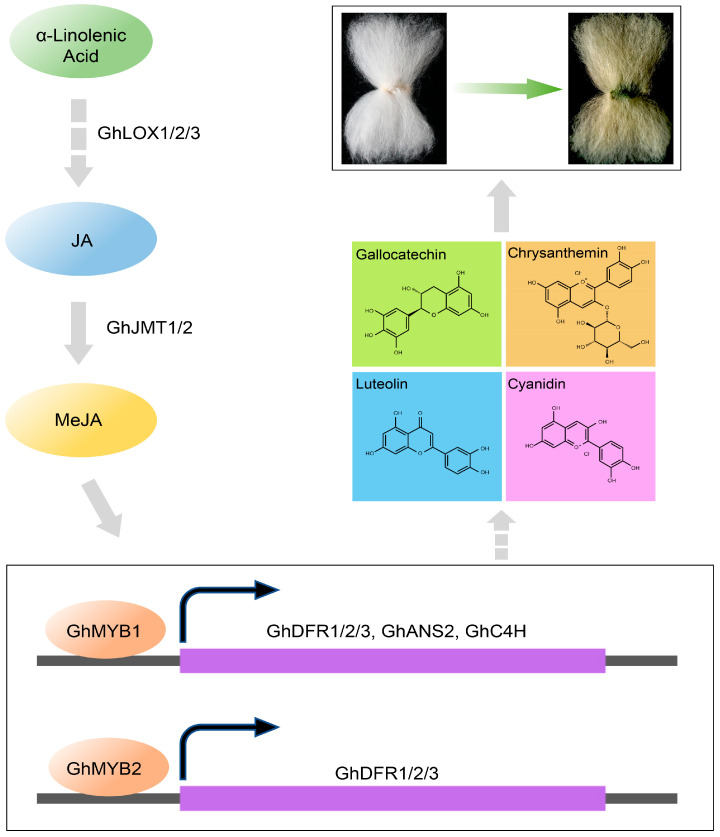
A hypothetical regulatory network schematic of TFs, flavonoid-related pathways genes, and flavonoid metabolites related to pigment deposition in GCFs via MeJA-mediated signaling. Each metabolite is represented with a different color background.

Research has demonstrated that MeJA-induced bHLH42 mediated the specific accumulation of anthocyanins in Caitai by regulating flavonoid metabolic pathways [20]. MeJA influences the synthesis of flavonoid metabolites by upregulating genes involved in the biosynthetic pathway (*PcCHS*, *PcCHI*, *PcF3H*, *PcDFR*, *PcANS*, and *PcLAR1*) in pear callus, with the involvement of MYBs [25]. In this study, a combined analysis of metabolomics and transcriptomics data revealed that *GhF3H*, *GhDFR1/2/3*, *GhANS1/2*, and *GhC4H* directly or indirectly regulate the synthesis of flavonoids including Luteolin, Gallocatechin, Nicotiflorin, Cyanidin, and Chrysanthemin (Figure 6). In MeJA-treated GCFs, the expressions of five flavonoid pathway genes, including *GhDFR1/2/3*, *GhANS2*, and *GhC4H,* were significantly increased (Figure 7A), leading to notable upregulation of four flavonoid metabolites (Figure 7B). These results infer that MeJA mediates the synthesis of flavonoid metabolites by inducing *GhMYB1/2*-regulated expression promotions of the flavonoid pathway genes *GhDFR1/2/3*, *GhANS2*, and *GhC4H*, thereby contributing to pigment synthesis and deposition in GCFs (Figure 8).

## 4. Materials and Methods

### 4.1. Plant Materials

Xincaimian7 (Cai7), a naturally colored cotton cultivar, was developed by the Xinjiang Natural Colored Cotton Research Institute through hybridization between upland cotton K202 (as the female parent) and colored upland cotton Lv2 (as the male parent), followed by six generations of pedigree selection. The green-colored cotton line Cai7 and the white cotton TM-1 were cultivated in the experimental fields of Shihezi University in 2023. Ovules and fibers from both Cai7 and TM-1 were collected for transcriptome sequencing at 0, 5, 10, 15, and 20 days post anthesis (DPA), with three biological replicates established for different times. The 1-DPA ovules were collected from the bolls and cultured on BT medium for 7 days, followed by 5 µM MeJA treatment, with the treated materials used for gene expression analysis (0, 3, 12, and 24 h; three biological replicates for each time point) and metabolite detection (0, 1, 3, 6, and 10 days; six biological replicates for each time point) for assessing metabolite accumulation. All samples were maintained at −80 °C.

### 4.2. RNA Extraction, Transcriptome Sequencing, and Analysis

Following the manufacturer’s operating procedures, RNA was extracted using the RNAprep Pure Plant Plus Kit (DP441, Tiangen Biochemical Technology, Beijing, China). High-quality RNA samples (RNA integrity number > 8.0) were subjected to sequencing analysis using the HiSeq 2000 sequencing system. The sequencing data underwent quality control, alignment, and expression quantification using the Trimmomatic+FastQC+Hisat2+Stringtie pipeline [35,36,37,38]. Detailed versions and parameters can be found in Shi et al. (2024) [1]. Transcript abundance was quantified using FPKM (fragments per kilobases of transcripts per million mapped reads) values [38], where the read sequences were aligned to the Upland Cotton TM-1 reference genome (CRI v1) [39]. Differential gene expression analysis was performed with DESeq2. The *p*-values were adjusted using the Benjamani–Hochberg (BH) correction method [40]. Genes with|Log2 (Fold change)| ≥ 1 and *FDR* < 0.05 were considered differentially expressed genes (DEGs) [1].

### 4.3. Transcription Factors Prediction, KEGG Enrichment, and PPI Network Construction

The protein sequences of the candidate DEGs were submitted to the Plant Transcription Factor Database (PlantTFDB v5.0) for batch prediction of transcription factors [41]. The Kyoto Encyclopedia of Genes and Genomes (KEGG) pathway enrichment analysis of the DEGs was performed using KOBAS3.0 (http://bioinfo.org/kobas/genelist/, accessed on 10 June 2024) [42]. PPI analysis was conducted using the STRING database (https://cn.string-db.org/, accessed on 10 June 2024) [43].

### 4.4. Quantitative Reverse Transcription PCR (RT-qPCR) Analysis

The extracted RNA was used to synthesize first-strand cDNA using the FastKing Reverse Transcription Kit (KR116, Beijing Tiangen Company, Beijing, China) according to the manufacturer’s instructions. For qPCR quantification, the reaction system was prepared with Novostar SYBR qPCR SuperMix Plus, and then the reaction was run on a Roche LightCycler 480 instrument. Data normalization was performed relative to expression of the GhUBQ7 reference gene [9]. Primer design was carried out using the online Primer-BLAST tool (http://www.ncbi.nlm.nih.gov/tools/primer-blast, accessed on 10 June 2024) [44].

### 4.5. Metabolome Analysis

The fibers of green cotton cultivar Cai7 and white cotton TM-1 were air-dried at room temperature for 30 days. Metabolites were extracted, identified, and quantitatively analyzed according to Liu et al. (2018) [9]. Metabolite variations between sample groups were assessed using variable importance in projection (VIP) scores derived from partial least squares discriminant analysis (PLS-DA). The criteria used to identify metabolites with statistically significant differences are defined as follows: (1) fold change ≥ 1.2, (2) VIP ≥ 1, and (3) *q* < 0.05 [45]. Clustering analysis was performed using the Tbtools-Heatmap module [46], calculating the Euclidean distance matrix of relative quantification values of metabolites and clustering with the complete linkage method.

### 4.6. HPLC-MS/MS Analysis

Luteolin, Gallocatechin, Nicotiflorin, Cyanidin, and Chrysanthemin were accurately weighed and dissolved in methanol to prepare a single standard solution at a concentration of 1.0 mg/mL. This solution was then diluted with methanol to create mixed standard solutions at concentrations of 1, 5, 10, 25, 50, 75, and 100 ng/mL. Chromatographic analysis was performed using an ACQUITY UPLC BEH C18 column (1.7 μm, 2.1 × 50 mm) at a temperature of 30 °C and a flow rate of 0.3 mL/min, with an injection volume of 1.0 μL. The optimized mobile phase comprised A: 0.1% formic acid in water and B: acetonitrile, employing a gradient elution program as follows: 0–3.0 min at 20–98% B, 3.0–4.5 min at 98% B, 4.5–5.0 min at 98–20% B, and 5.0–6.0 min at 20% B. Detection was conducted using an electrospray ion source with a source temperature of 150 °C, capillary voltage of 3.0 kV, desolvation gas temperature of 450 °C, desolvation gas flow rate of 800 L/h, cone gas flow rate of 150 L/h, and nebulizer pressure of 7.0 Bar, utilizing multiple reaction monitoring (MRM) mode.

### 4.7. Statistical Analysis

A Student’s *t*-test was used to process the significance analysis of RT-qPCR, the determination of widely targeted metabolite content, and HPLC-MS analysis results [47]. The significance levels were as follows: * *p* less than 0.05, ** *p* less than 0.01, *** *p* less than 0.001, and NS means not significant. Statistical analyses were conducted using GraphPad Prism (version 8.01, GraphPad Software, San Diego, CA, USA).

## 5. Conclusions

By integrating multi-omics (transcriptomics and metabolomics) data with biochemical characterization in GCFs, we conclude that MeJA might act as an important regulator involved in pigment deposition in GCFs, via mediating the MYB-regulated levels of genes and metabolites of the flavonoid pathway. Our discovery provides a molecular basis for enhancing the stability and strength of natural colored cotton through genetic engineering. These strategies not only meet the needs of sustainable development in the textile industry but also accelerate their practical applicability.

## Data Availability

The datasets generated and/or analyzed during this study are deposited in the [NCBI Gene Expression Omnibus (GEO)] repository (Accession number GSE76400), [https://www.ncbi.nlm.nih.gov/geo/query/acc.cgi?acc=GSE76400&_refluxos=a10] accessed on 10 June 2024.

## Supplementary Materials

The following supporting information can be downloaded at: https://www.mdpi.com/article/10.3390/genes16050599/s1. Table S1. Primers used for RT-qPCR. Figure S1. Pathway diagram of UDEGs in the α-linolenic acid metabolism pathway of Cai7 vs. TM-1 transcriptome. Figure S2. Pathway diagram of UDEGs in the phenylpropanoid biosynthesis pathway of Cai7 vs. TM-1 transcriptome. Additional file 4: Figure S3. Pathway diagram of UDEGs in the flavonoid biosynthesis pathway of Cai7 vs. TM-1 transcriptome. Figure S4. Pathway diagram of UDEGs in the flavone and flavonol biosynthesis pathway of Cai7 vs. TM-1 transcriptome. Figure S5. Pathway diagram of UDEGs in the α-linolenic acid metabolism pathway of different developmental periods of Cai7 fibers. Figure S6. Pathway diagram of UDEGs in the phenylpropanoid biosynthesis pathway of different developmental periods of Cai7 fibers. Figure S7. Pathway diagram of UDEGs in the flavonoid biosynthesis pathway of different developmental periods of Cai7 fibers. Figure S8. Pathway diagram of UDEGs in the flavone and flavonol biosynthesis pathway of different developmental periods of Cai7 fibers. Figure S9. Overview of metabolite samples.

## Abbreviations

The following abbreviations are used throughout this manuscript:

*NCC*	Naturally colored cotton
*GCFs*	Green cotton fibers
*DPA*	Days post anthesis
*TF*	Transcription factor
*DEGs*	Differentially expressed genes
*UDEGs*	Upregulated differentially expressed genes
*CUDEGs*	Co-expression upregulated differentially expressed genes
*MeJA*	Methyl jasmonate
*KEGG*	Kyoto Encyclopedia of Genes and Genomes
*PPI*	Protein–protein interaction
*RT-qPCR*	Reverse transcription quantitative polymerase chain reaction
*FPKM*	Fragments per kilobase of transcript per million mapped reads
*VIP*	The variable importance in projection
*PLS-DA*	The partial least squares discriminant analysis
