# Supplementary material for: Combined Analysis of Transcriptomes and Metabolomes Reveals That MeJA-Mediated Flavonoid Biosynthesis Is Crucial for Pigment Deposition in Naturally Colored Green Cotton Fibers
Source: Genes (Basel). 2025 May 19;16(5):599. doi: 10.3390/genes16050599 (PMC12110993; doi:10.3390/genes16050599)
Supplement: Supplementary file 1 [file genes-16-00599-s001.zip › genes-3644918-supplementary.pdf]

Table S1. Primers used for RT-qPCR.

Primer	Sequence(5'-3')
qGhC4H-F1	ATCCCTGCAGAGAGTAAGAT
qGhC4H-R1	TCCCATTTGCTTCTACCTTC
qGhDFR1-F1	CTTGTGCAGAGCTCATATCT
qGhDFR1-R1	TCGATAATGGTAGCATGGTG
qGhANS1-F1	AGTGCACTCACTTTCATTCT
qGhANS1-R1	TGCATGATGATGGAGTTAGG
qGhANS12-F1	AGTGCACTCACTTTCATTCT
qGhANS12-R1	GAACGTATTTCGCGGTAATC
qGhF3H-F1	AGATTGTCAATAGCCACGTT
qGhF3H-R1	GCTCCTCAAGGATTGGTTTA
qGhDFR2-F1	TCGATCATAAAACAAGGCCA
qGhDFR2-R1	CGATAATGGTAGCATGGTGA
qGhDFR3-F1	CTGTGGAGACATGTCAAGAA
qGhDFR3-R1	ACACTGTTTGGTGTCTCAAT
qGhMYB2-F1	GGATGAATTATCTCCGACCC
qGhMYB2-R1	CTTCTTCATCGGGAGCAATA
qGhMYB1-F1	AAGCTATTAGGTGTAGCAGC
qGhMYB1-R1	ATACCACCACCCATTTCTTC
qGhLOX1-F1	GCCGACCGGCTTCTAAAATA
qGhLOX1-R1	AAGTGCTTTCAACCTCCCAG
qGhLOX3-F1	TTGAGCACAACCTAGAGGGA
qGhLOX3-R1	GCAGGTACGGCATAAGTGAA
qGhJMT1-F1	ATCCCACCACCGAAAATAGC
qGhJMT1-R1	CCTCAGTGCATGGAGCATAG
qGhJMT2-F1	CATACTATGCTCCATGCGCT
qGhJMT2-R1	ATGAGGGGTATGGGTATCGG
qGhLOX2-F1	TGCCAAAGCCTCAAGAACAT
qGhLOX2-R1	TGGTTGGTGATGAGAACAGC

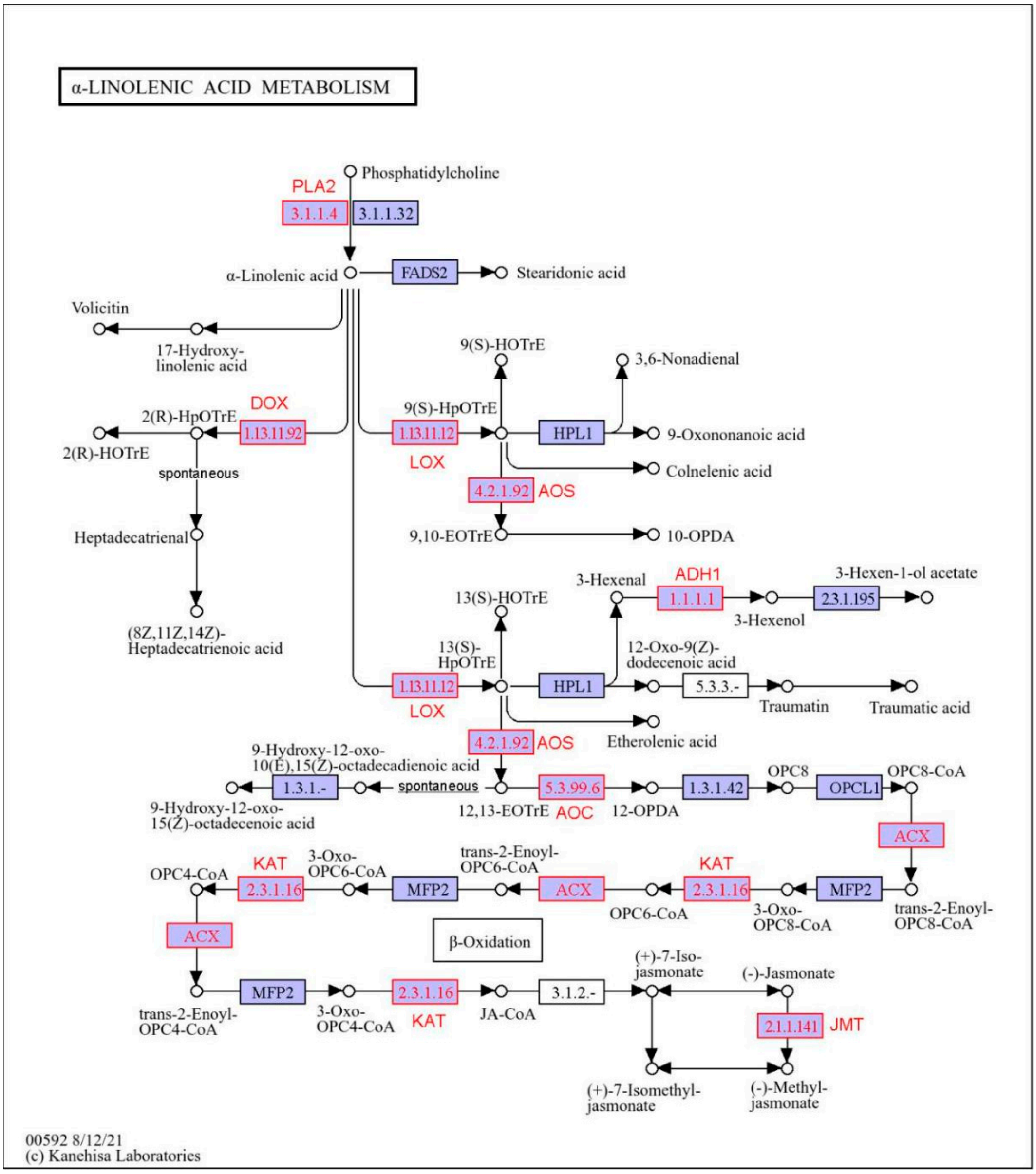

Figure S1. Pathway diagram of UDEGs in the α-linolenic acid metabolism pathway of Cai7 vs TM-1 transcriptome. Red texts and numbers indicate the UDEGs of α-Linolenic acid metabolism pathway involved in MeJA synthesis.

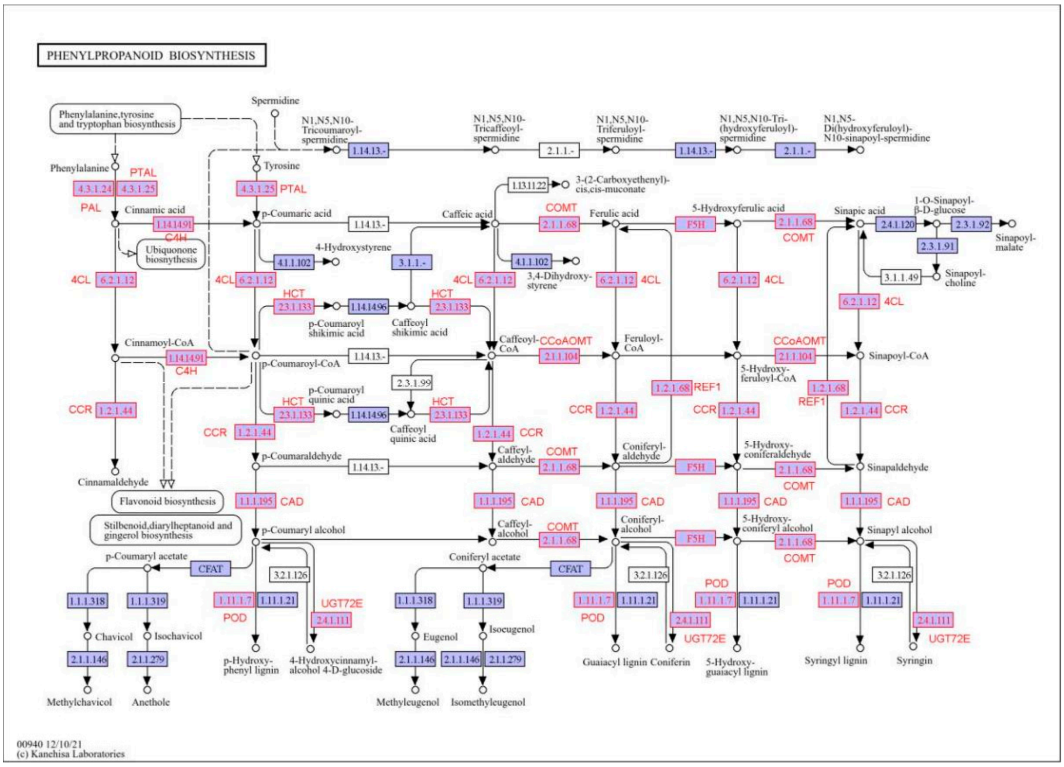

Figure S2. Pathway diagram of UDEGs in the phenylpropanoid biosynthesis pathway of Cai7 vs TM-1 transcriptome. Red texts and numbers indicate the UDEGs involved in phenylpropanoid biosynthesis.

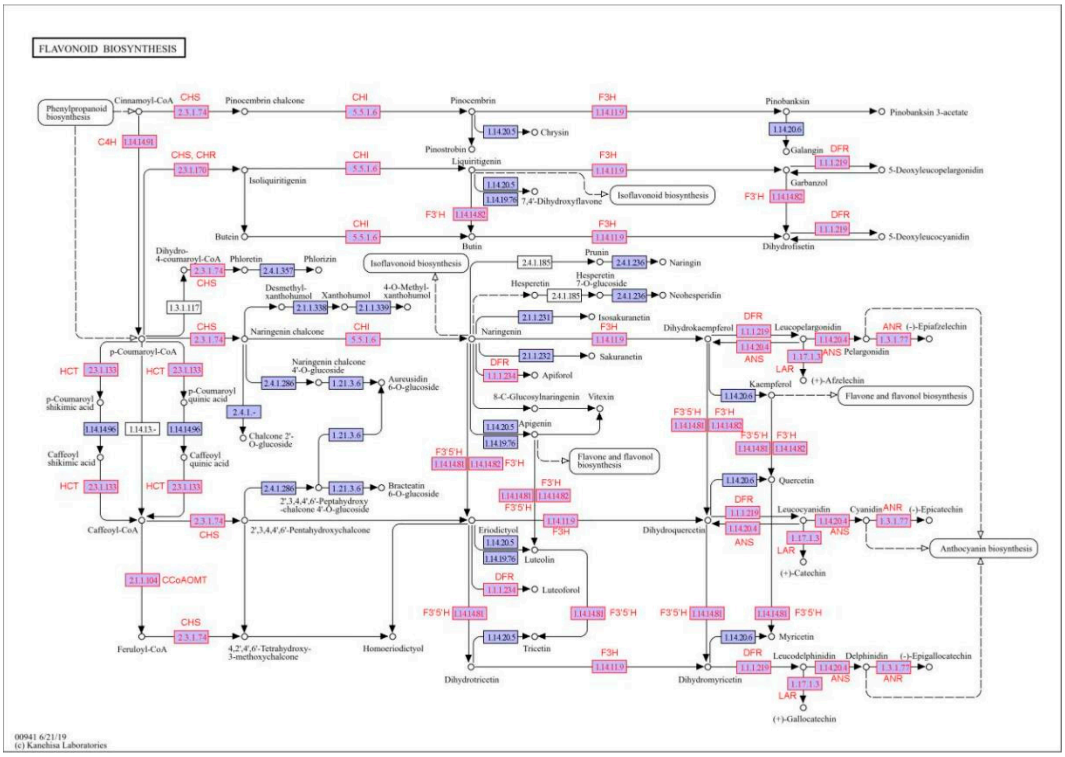

Figure S3. Pathway diagram of UDEGs in the flavonoid biosynthesis pathway of Cai7 vs TM-1 transcriptome. Red texts and numbers indicate the UDEGs involved in flavonoid biosynthesis.

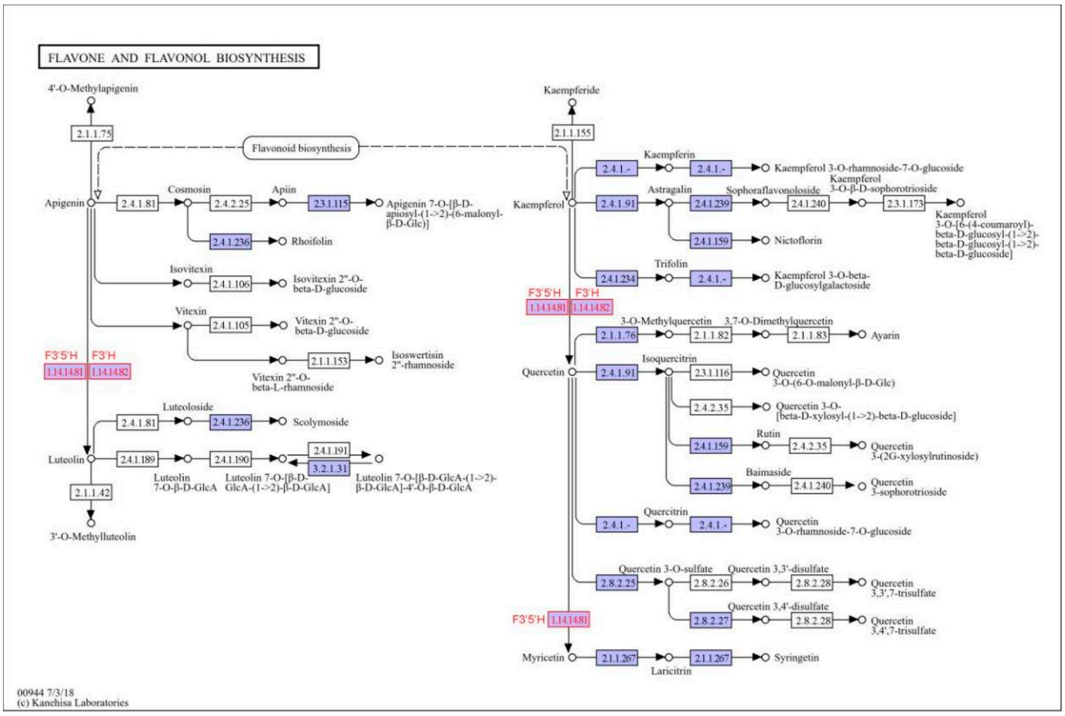

Figure S4. Pathway diagram of UDEGs in the flavone and flavonol biosynthesis pathway of Cai7 vs TM-1 transcriptome. Red texts and numbers indicate the UDEGs involved in flavone and flavonol biosynthesis.

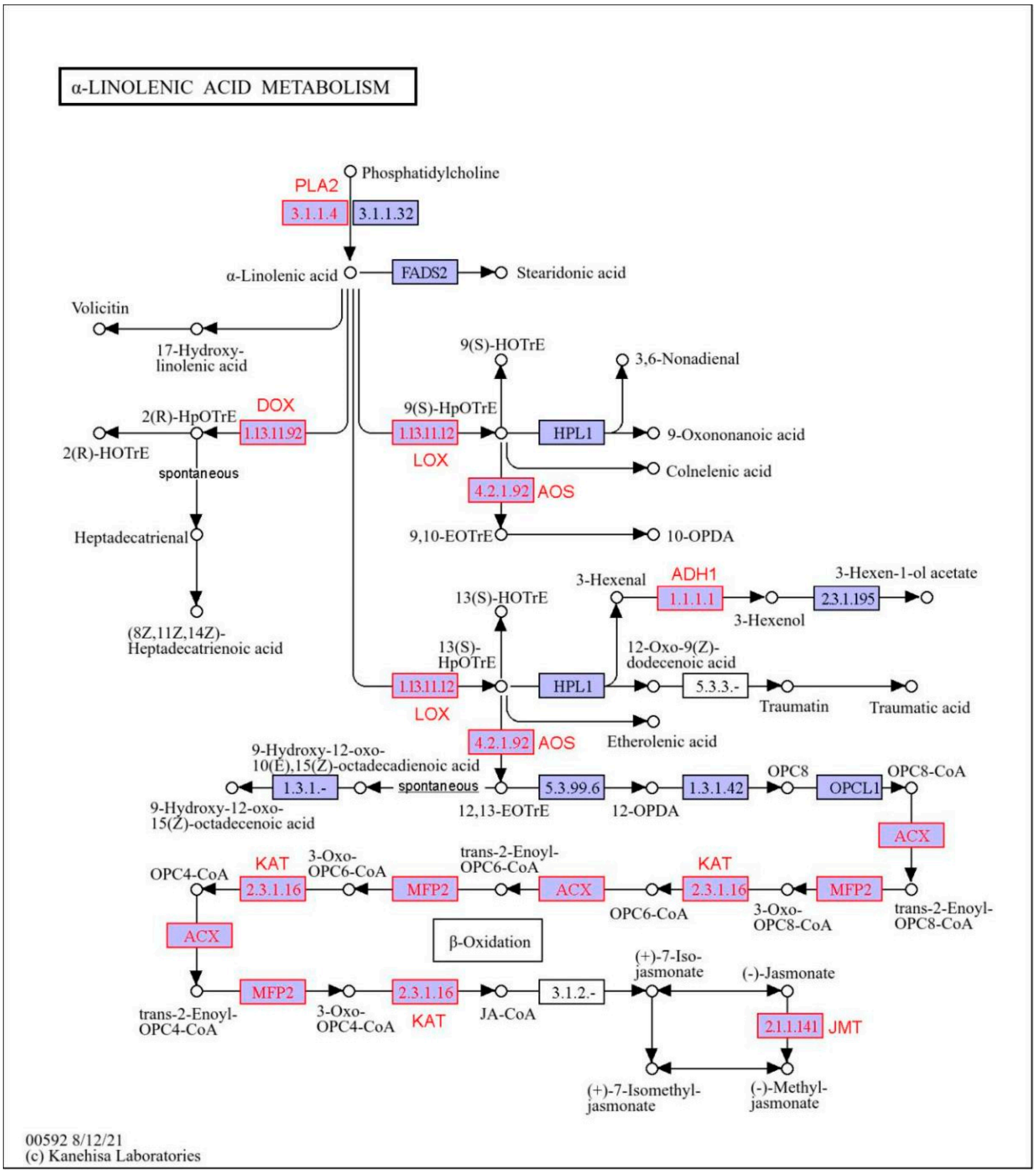

Figure S5. Pathway diagram of UDEGs in the α-linolenic acid metabolism pathway of different developmental periods of Cai7 fibers. Red texts and numbers represent the UDEGs of α-Linolenic acid metabolism pathway involved in MeJA synthesis.

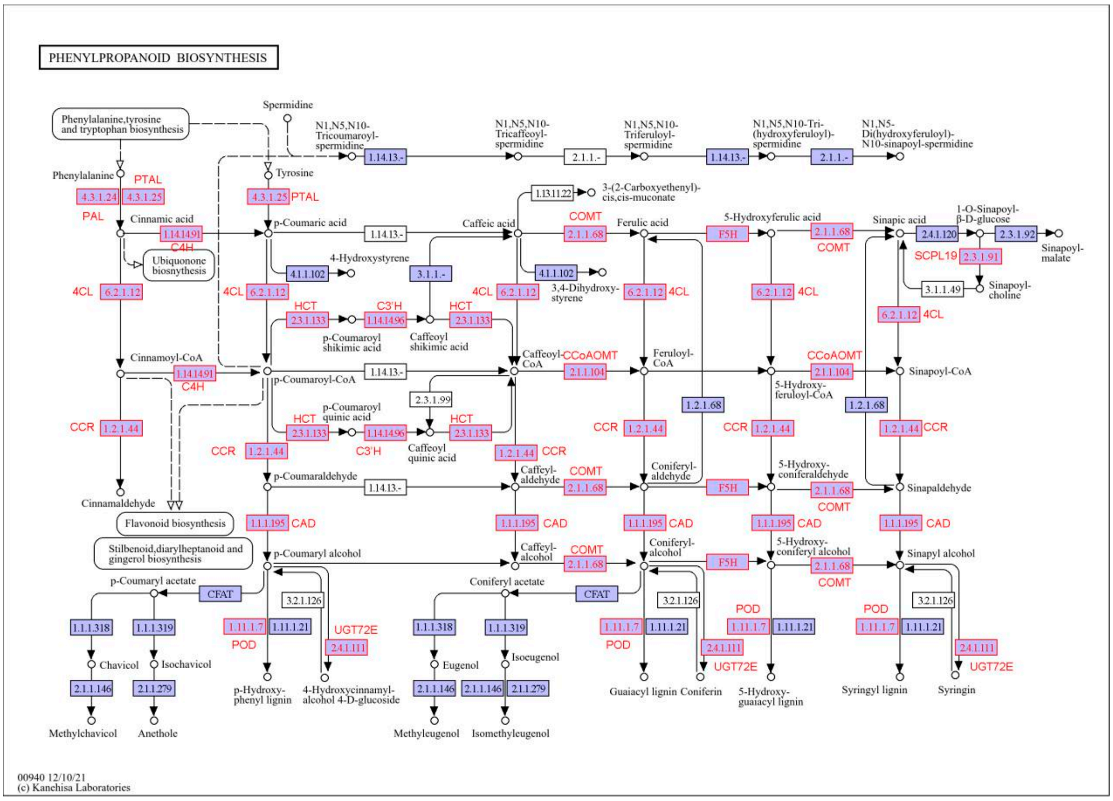

Figure S6. Pathway diagram of UDEGs in the phenylpropanoid biosynthesis pathway of different developmental periods of Cai7 fibers. Red texts and numbers represent the UDEGs involved in phenylpropanoid biosynthesis.

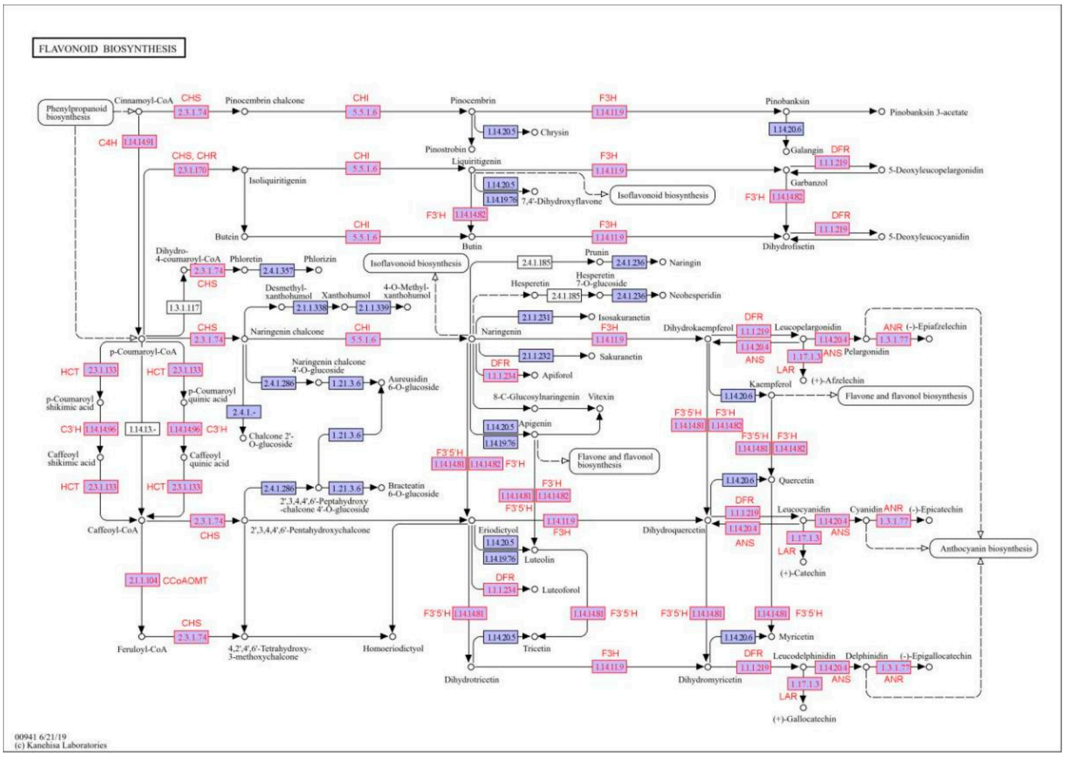

Figure S7. Pathway diagram of UDEGs in the flavonoid biosynthesis pathway of different developmental periods of Cai7 fibers. Red texts and numbers represent the UDEGs involved in phenylpropanoid biosynthesis.

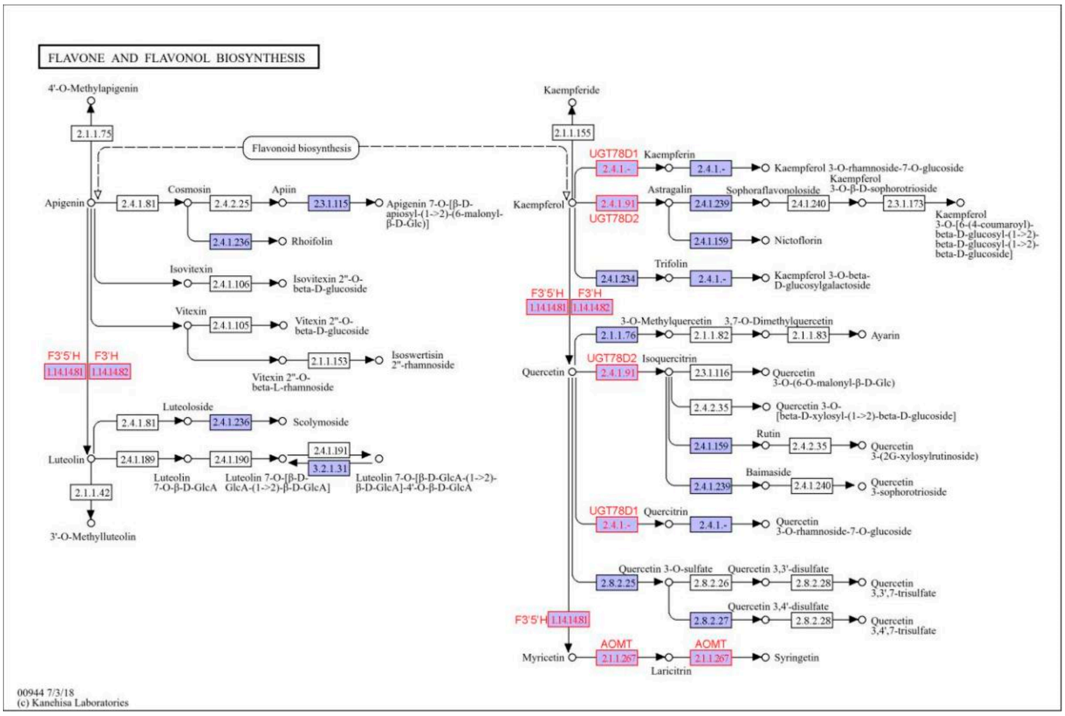

Figure S8. Pathway diagram of UDEGs in the flavone and flavonol biosynthesis pathway of different developmental periods of Cai7 fibers. Red texts and numbers represent the UDEGs involved in flavone and flavonol biosynthesis.

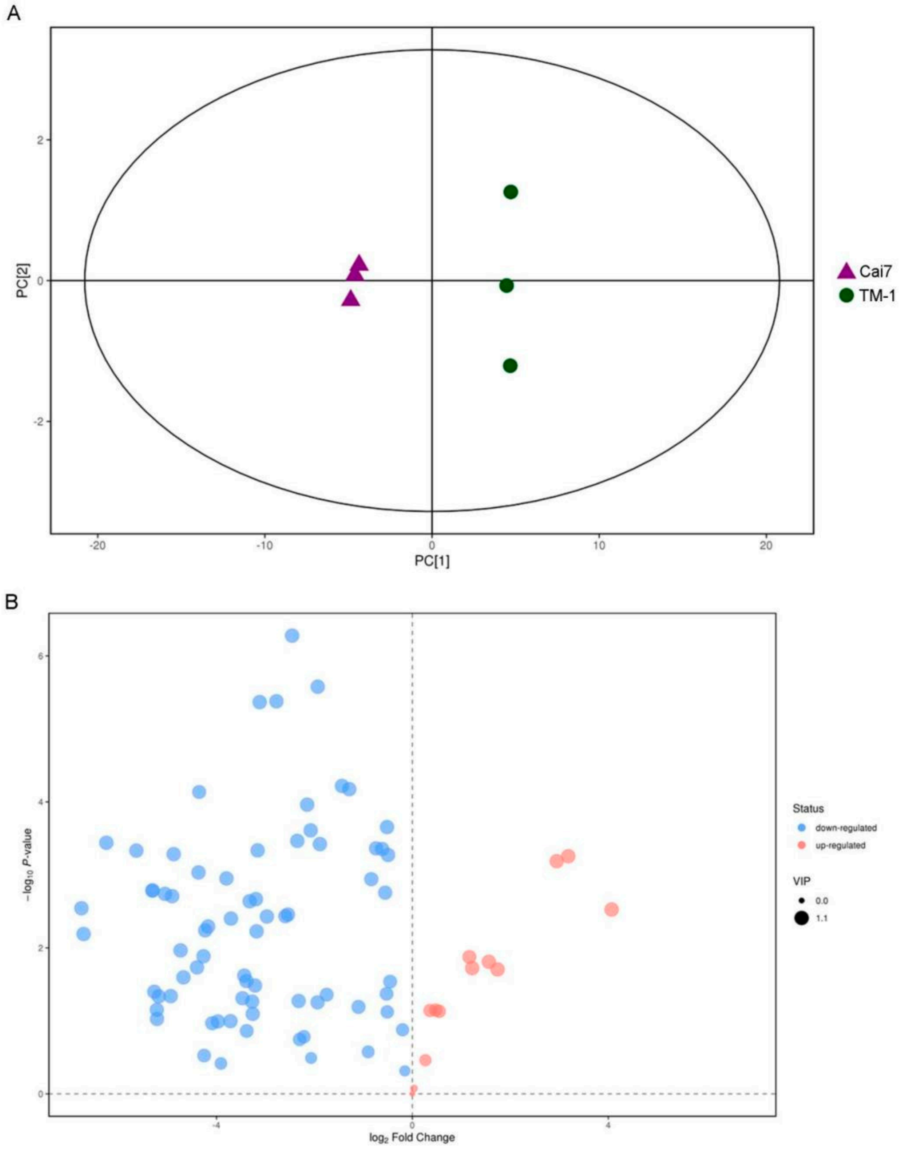

Figure S9. Overview of the metabolite samples. (**A**) PCA analysis of metabolic samples from TM-1 and Cai7. (**B**) The differential changes of all flavonoid metabolites.
